# Determination of Rose Alcohol Composition in Extracts and Flowers via Headspace Solid-Phase Microextraction and GC-MS

**DOI:** 10.1021/acsmeasuresciau.5c00112

**Published:** 2025-11-04

**Authors:** Amber M. Hupp

**Affiliations:** 8717 The College of the Holy Cross, 1 College St, Worcester, Massachusetts 01610, United States

**Keywords:** rose fragrance, rose alcohols, gas chromatography mass spectrometry (GC-MS), principal component analysis (PCA), headspace solid-phase microextraction (HS-SPME)

## Abstract

Natural extracts of rose are frequently subject to adulteration due to their high production costs and strong demand within the flavor and fragrance industry. Gas chromatography coupled with mass spectrometry (GC-MS) serves as a critical analytical tool for detecting adulterants and quantifying the relative concentrations of key rose alcohols. In this study, rose fragrances were analyzed by using GC-MS to assess compositional integrity. Standard accords consisted of six synthetic compounds with varying molecular weights, boiling points, and polarities, commonly found in rose essential oils and perfumes. Each component was baseline resolved on a nonpolar column and detectable at low concentrations (3–5%). Calibration curves were developed for phenyl ethyl alcohol, citronellol, and geraniol based on representative concentrations in commercial samples. Principal component analysis (PCA) was performed to further characterize and differentiate the accords according to their chemical profiles. Additionally, headspace solid-phase microextraction (HS-SPME) was used to sample the volatile profile of each sample on a fragrance strip. Several commercial samples, including two natural rose extracts (absolute and oil) and a commercial perfume, and a peony flower, were analyzed using liquid direct injection and/or HS-SPME. These results demonstrate the utility of GC-MS and HS-SPME for both quantitative and qualitative analysis of rose alcohols in complex fragrance matrices.

## Graphical abstract

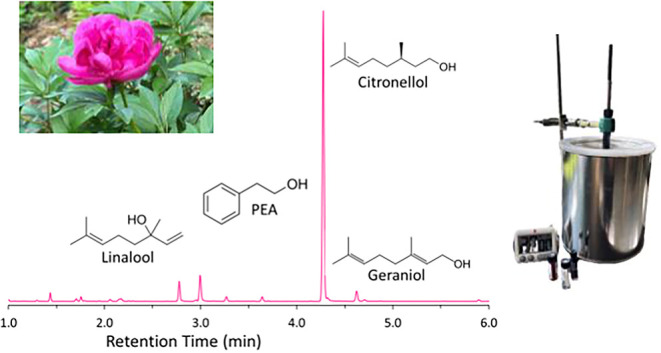

Roses are among the most widely recognized and esteemed flowers. Depending on their color, variety, and quantity in a bouquet, roses can convey sentiments of love, friendship, beauty, and opulence. The genus *Rosa* comprises over one hundred species and as many as 20 thousand distinct varieties. Celebrated for both their aesthetic appeal and fragrance, it is unsurprising that considerable effort has been devoted to cultivating new hybrid varieties that enhance growing season, improve resilience to colder climates, introduce novel color patterns, or increase oil yield.^1^Natural rose-derived materials, such as rose essential oil and rose absolute, as well as synthetic compounds, are fundamental components within the fragrance portfolios of major cosmetic companies and fragrance houses. Although numerous rose species exist, only a select few, most notably *Rosa damascena* and *Rosa centifolia*, are employed in the fragrance industry for natural material production due to their high oil yield upon extraction.^2^

Natural rose materials rank among the most costly natural floral materials on the market.^3^ The price of the oil or extract is closely linked to the quality of the material, with premium products being both undiluted and unadulterated.^2^ Natural rose materials are typically characterized by the relative concentrations of key rose alcohols: phenyl ethyl alcohol (PEA), citronellol, and geraniol, as well as the presence of additional molecular constituents. Rose essential oil, the most prized of rose-derived naturals, is obtained via the steam or water distillation of rose petals. Rose concrete is produced through hexane extraction, and its alcohol soluble fraction is referred to as rose absolute.^2^ Rose oil generally contains a low percentage of PEA (0.88–2.2%) and higher levels of citronellol and geraniol (26–62%).^4–8^ In contrast, rose absolute exhibits an inverse composition, with a high concentration of PEA (50–79%) and lower levels of citronellol and geraniol (6.5–33%).^6,9^ This variation arises from the water solubility of PEA. The composition of each oil or absolute depends on such factors as cultivation region, species, and extraction methodology (e.g., solvents, temperatures).^2,9^ Due to its high production cost and strong market demand, rose oil is frequently adulterated with less expensive materials, including vegetable oils or synthetic analogs of its natural constituents (e.g., synthetic PEA).

With the availability of routine synthetic methods, some fragrance houses will opt to utilize entirely synthetic accords that replicate the desired olfactory profile of rose. These accords typically include synthetic versions of the three rose alcohols in varying proportions, along with other compounds found in natural rose (e.g., eugenol, linalool, rose oxide) or those that contribute specific aromatic facets (e.g., dimethyl acetaldehyde phenyl acetate (PADMA) or cis-3-hexenal for a green note). Such accords may also incorporate natural oils or isolated molecules (isolates) from natural oils. Many of these components have been studied for their olfactory contributions to rose accords.^10^

The most commonly employed instrumental technique for determining the composition of volatile fragrance compounds is gas chromatography, typically coupled with Mass Spectrometry (GC-MS). In this technique, fragrance oils and absolutes are diluted in an organic solvent and analyzed for both qualitative identification of chemical composition and quantitative determination of concentration. GC-MS is widely used in the literature for analyzing rose oils and absolutes, and although proprietary methods are rarely published, GC-MS remains the preferred instrumental technique in major fragrance houses.^4,5,8,10,11^

As with all volatile mixtures, the headspace vapor above a liquid exhibits a different component ratio than the liquid itself, in accordance with Raoult’s Law. Variations in the vapor pressure cause certain components to be either enriched or diminished in the vapor phase. This phenomenon has prompted interest in analyzing the headspace of fragrance materials. A widely adopted method for such analysis is solid-phase microextraction (HS-SPME).^12^ In this approach, a specialized fiber is exposed to the vapor phase above a liquid or solid sample, where volatile compounds adsorb onto the fiber’s surface. The fiber is then inserted into the GC-MS injection port, where the volatiles are thermally desorbed and introduced into the chromatographic column. HS-SPME has proven effective for profiling the aroma of rose alcohols in flowers and other fragrant materials.^13–15^ Moreover, HS-SPME is valuable for analyzing solid samples that are otherwise difficult to assess via conventional GC-MS. Examples include fresh roses on the bush, freshly cut flowers, and commercial products such as lotions and candles.^3,9^ Most published HS-SPME studies examined samples in isolation, either the oil or the headspace, the flower or the oil, or the oil versus the absolute, rather than analyzing both simultaneously. Consequently, methodologies vary across research groups. Furthermore, no published data currently address how the composition of rose alcohols may differ from the liquid oil or absolute to its corresponding headspace of the oil or absolute or between the flower and the resulting extract.

This study presents a method for analyzing both liquid and headspace rose samples applicable throughout the production process of commercial rose materials. The objectives of this experiment are (1) to develop a method for quantifying rose alcohols in liquid products (naturals and synthetics); (2) to establish a technique for identifying volatile components via headspace sampling of liquids (oils, absolutes, perfumes) and solid materials (scent strips, flowers); and (3) to investigate the influence of extraction time on the analysis of rose alcohols in the vapor phase. A unified method applicable across all material types, from the rose flower to the natural oil product to the final fragrance product represents a significant advancement in detecting adulteration and assessing rose alcohol content in situ and has not been previously documented.

## Materials and Methods

### Materials

Perfumers quality phenyl ethyl alcohol (PEA), damascene β, ionone β, and eugenol were obtained from a raw aromatic and flavor materials provider (Perfumers Apprentice, Scotts Valley, CA), while geraniol (98%, Aldrich) and citronellol (95%, Acros Organics) were obtained from chemical supply companies. Various physical properties and chemical structures for each component are listed in Table 1. Ethanol (200 proof, Decon Laboratories) was utilized for all dilutions and extractions. Fragrance test strips were purchased from Orlandi and, after being sprayed, were cut to fit inside each vial for SPME analysis. Appropriate safety protocols for handling irritants and flammables were in place throughout the experiment.

**Table 1:**
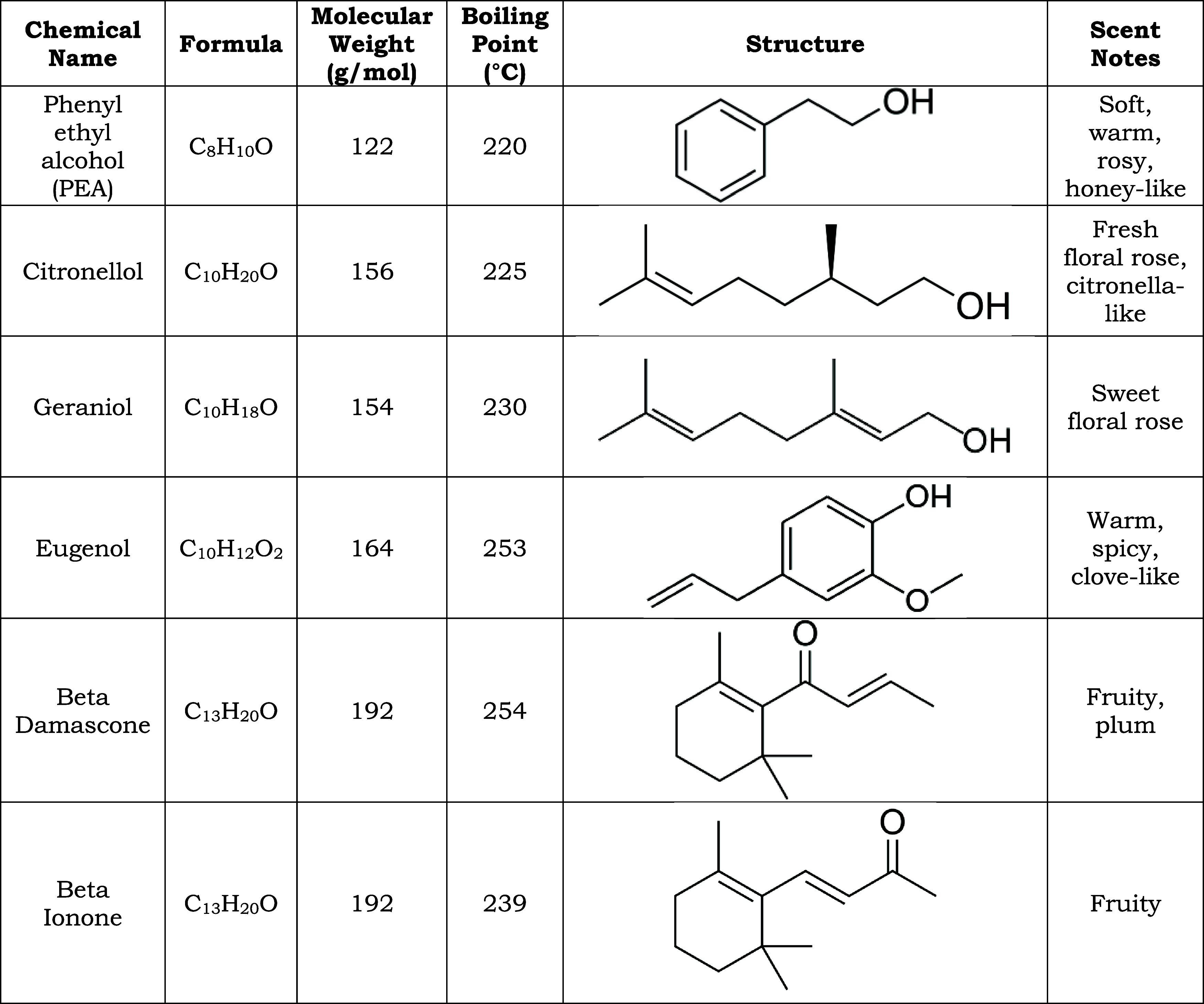
Physical Properties of Common Chemical Components in Natural Rose Extracts and Perfumes

Rose accord standards were created by dropwise addition (v/v) according to Table 2. For liquid injection, the rose standards were diluted as a 10% (w/w) solution in ethanol. For headspace analysis by SPME, the standards were sampled by dipping the fragrance test strip (approximately 2.5–3 cm) into the undiluted rose accord, then placing in a vial and sealing almost immediately.

**Table 2:** Composition (%v/v) of Rose Accord Standards, Labeled A through L

	dilution % of stock	A	B	C	D	E	F	G	H	I	J	K	L
phenyl ethyl alcohol	10	34	50	60	26	12	34	34	34	34	34	34	34
citronellol	10	34	26	12	50	60	34	34	34	34	34	34	34
geraniol	10	9	9	9	9	9	4	4	4	15	9	9	9
eugenol	10	3	3	3	3	3	3	3	3	3	3	3	0
β damascone	1	5	5	5	5	5	5	5	5	5	5	0	5
β ionone	10	5	5	5	5	5	5	9	0	5	0	5	5
ethanol		10	2	6	2	6	15	11	20	4	15	15	13
total		100	100	100	100	100	100	100	100	100	100	100	100

A commercial rose perfume (L’EXTASE Rose Absolue Eau du Parfum by Nina Ricci) and commercial rose extracts (Perfumers Apprentice, absolute, and oil) were tested. For liquid injection, the rose samples were diluted as a 10% (w/w) solution in ethanol. For headspace analysis by SPME, the perfume was sprayed twice directly onto a scent strip, while the natural rose extracts were sampled by dipping a strip into the undiluted liquid. The scent strips were placed immediately into a 20 mL vial equipped with a septum cap. To test the method with a botanical, a peony flower was placed inside a clean paint can with a small hole in the lid for introducing the SPME fiber.

Headspace samples were analyzed the same day, usually directly after sampling by SPME. The SPME fiber was composed of 50/30 μm divinylbenzene/Carboxen on PDMS on a StableFlex fiber (Supelco). This type of SPME fiber was chosen for its ability to extract volatile, low-molecular-weight compounds across a variety of polarities, making it an excellent choice for aroma molecules. Extractions were performed at ambient temperature for 20 min, unless otherwise specified. The fibers were thermally desorbed for 1.0 min in the injection port (at 250 °C) directly prior to analysis. Complete desorption of all analytes occurred in 1.0 min, which was determined through repeat desorption experiments where no carryover was observed. Therefore, no additional conditioning was needed for the SPME fiber prior to the subsequent analysis.

### Instrumentation

Separations were performed by using an Agilent 6890 gas chromatograph coupled with an Agilent 5973 mass spectrometer (Agilent Technologies, Santa Clara, TX). The GC was equipped with a nonpolar ZB-5 column (5% phenyl polydimethylsiloxane, Phenomenex, 30 m × 0.25 mm × 0.25 μm i.d.). The oven temperature was optimized for separation of the six components in the rose accord as follows: 100 to 200 °C at 10 °C/min, yielding a total run time of 10.0 min. High purity helium was used as a carrier gas at a flow rate of 0.8 L/min. Each liquid sample was manually injected (1 μL from 10 μL syringe, Hamilton Company) with a split ratio of 200:1. Each headspace sample was desorbed from the SPME fiber by using a split ratio of 50:1. The inlet and transfer line temperatures were held at 250 and 280 °C, respectively. An electron-impact ionization source was utilized with a quadrupole mass analyzer operated in full-scan mode (*m*/*z* 40 – 600) with a sampling rate of 2.6 scans/s. The mass spectrometer source and quadrupole were held at 230 and 150 °C, respectively.

Peak identification was performed using a NIST database (NIST14, Gaithersburg, MD) as well as retention time and mass spectral comparisons to external standards. The area of each peak was identified via integration using a common threshold (Enhanced Chemstation D.03.00.611, Agilent). For chemometric analysis, peak areas were normalized using the total area under the chromatogram in Microsoft Excel 2016. Normalized peak areas were mean-centered in Pirouette 4.5 (Infometrix, Bothell, WA) prior to subsequent chemometric analysis. All figures were created using the plotting feature in Microsoft Excel.

## Part 1. Analysis of Rose Accords

The standard rose accords were analyzed by using GC-MS. A representative chromatogram for one rose standard, obtained via direct liquid injection, is shown in Figure 1. Each component is baseline resolved with a sufficient signal-to-noise ratio, including β damascene, which is present at 0.05% in the rose accord. The elution order is primarily determined by molecular weight and boiling point, as listed in Table 1, with some influence from polarity due to the 5% phenyl column.

**Figure 1:**
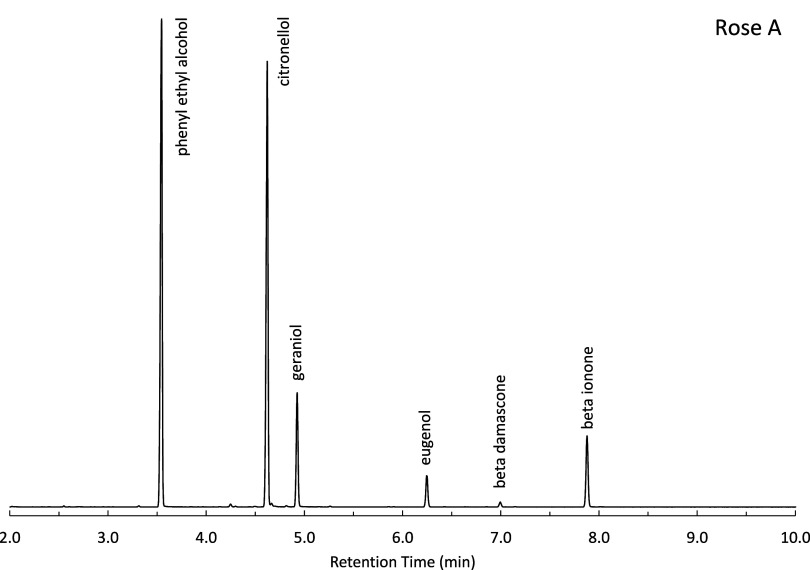
Chromatogram of rose standard A, direct injection of liquid.

To better characterize the standard set in Table 2, PCA was performed by using normalized peak areas for the six components. Scores plots for PC1 and PC2 are shown in Figure 2A, while the plot for PC3 is shown in Figure 2B. Across PC1 (which accounts for 91.5% of the variation), rose standards are clustered based on differences in PEA and citronellol concentration; samples with high PEA and low citronellol appear on the left (negative loadings), while those with low PEA and high citronellol appear on the right (positive loadings).

**Figure 2:**
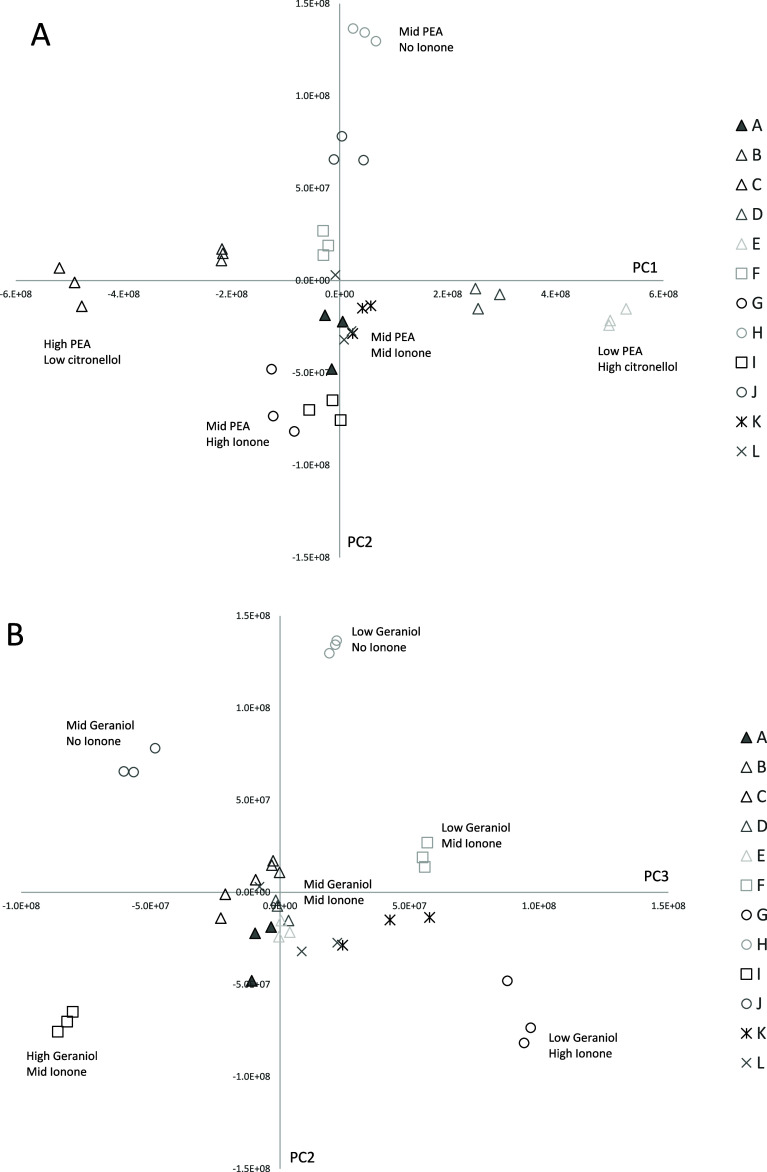
PCA of rose standards. Figure 2A displays PC1 versus PC2, while Figure 2B displays PC2 versus PC3. Samples are grouped according to the concentration of each component. Triangles with their various shading show samples A-E where PEA and citronellol concentration vary. For example, sample C, black triangles, has a relatively high concentration of PEA and a relatively low concentration of citronellol. Squares show differences in geraniol, circles show differences in β ionone, and stars show differences in eugenol and β damascene.

In PC2 (5.1%), samples are differentiated by the differences in β ionone content. Rose standards lacking β ionone load positively on PC2, whereas those with higher β ionone concentrations load negatively. PC3 (3.2%) reflects variations in the geraniol concentration. When PC2 and PC3 are plotted together, as shown in Figure 2B, distinct regions emerge where samples are categorized according to both their geraniol and β ionone concentrations.

The loadings for each PC (not shown) correspond to the chemical differences described above. Accordingly, fragrance standards are grouped based on their unique chemical compositions, thus demonstrating the potential of PCA to distinguish between different types of fragrance samples, even within the same general fragrance class (i.e., rose). With a larger data set encompassing greater sample variability, PCA could yield further insights into chemical composition.

Standards A-I exhibited varying rose alcohol compositions, as shown in Table 2. The resulting percent area of each component (calculated as the peak area divided by the total area under all peaks from each chromatogram) is shown in Figure 3. Since these standards were analyzed via direct liquid injection, the percent area of each peak accurately reflects the original composition. Standard deviations (*n* = 3) are displayed graphically on each bar and ranged from 0.001% for compounds at low concentrations (e.g., β damascene) to 1.7% for those at higher concentrations (e.g., PEA and citronellol). These results indicate that the liquid injection method is highly reproducible and reliably representative of the original liquid sample, as expected.

**Figure 3:**
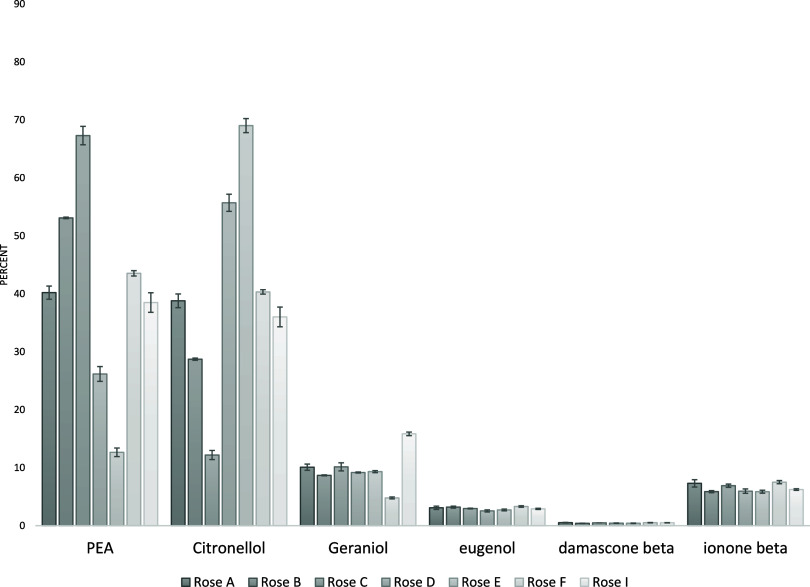
Percent area of each component in the rose standards using direct liquid injection

The percent composition of each rose alcohol was used to generate a calibration curve based on the volume percentage. Standards A-E were used for PEA and citronellol (12–60%), while standards A-I were used for geraniol (4–15%). These curves represent the normalized area as a function of percent by volume of each rose alcohol, with the resulting regression shown in Table 3. The linear fits, as indicated by *R* ^2^, demonstrate strong correlations, confirming a linear relationship between the percent composition of each rose alcohol and the normalized area for each peak in the chromatogram. This approach suggests that quantitation can be achieved using percent area alone, without the need for an internal standard.

**Table 3:** Calibration Information for Rose Standards through Direct Injection^a^

	regression	*R* ^2^	lab rose	rose absolute	rose oil	rose parfum
PEA	*y* = 1.437 × 10^7^ × – 1.368 × 10^7^	0.990	36 ± 1	51	45	6*
citronellol	*y* = 1.493 × 10^7^ × – 2.147 × 10^7^	0.999	37 ± 1	22	5	4*
geraniol	*y* = 1.281 × 10^7^ × + 1.118 × 10^7^	0.999	13 ± 1	11	0.4*	0.9*

a Percent (%) composition for each “unknown” sample are provided. Those percent compositions labeled with * fall outside the calibration range of the regression.

Next, commercial samples of rose extracts and perfumes were analyzed, and the percent composition of each rose alcohol was determined using the calibration curves described above. The resulting percent compositions are listed in Table 3. The lab-created rose was formulated with a composition of 38 parts PEA, 38 parts citronellol, and 8 parts geraniol, with the remaining three components (eugenol, β damascene, and β ionone) completing the total to 100 parts by volume. This method demonstrates strong predictive accuracy for percent composition in the lab-created sample. Standard deviations (*n* = 3) for the lab rose sample, obtained via direct injection, are reported in Table 5. These deviations are minimal, and the true values are just outside the confidence intervals, indicating the high reproducibility and reliability of the analytical method.

Chromatograms illustrating the separation of rose oil and absolute rose oil are shown in Figure 4. Rose absolute, a dark yellow-orange, transparent, slightly viscous liquid, primarily contains the three rose alcohols, as the petals are not exposed to heat during the extraction process. In contrast, rose oil, a pale yellow liquid, contains a broader range of additional components due to the use of heated solvent extraction. The compositions of both rose absolute and rose oil are consistent with traditional profiles for these sample types.

**Figure 4:**
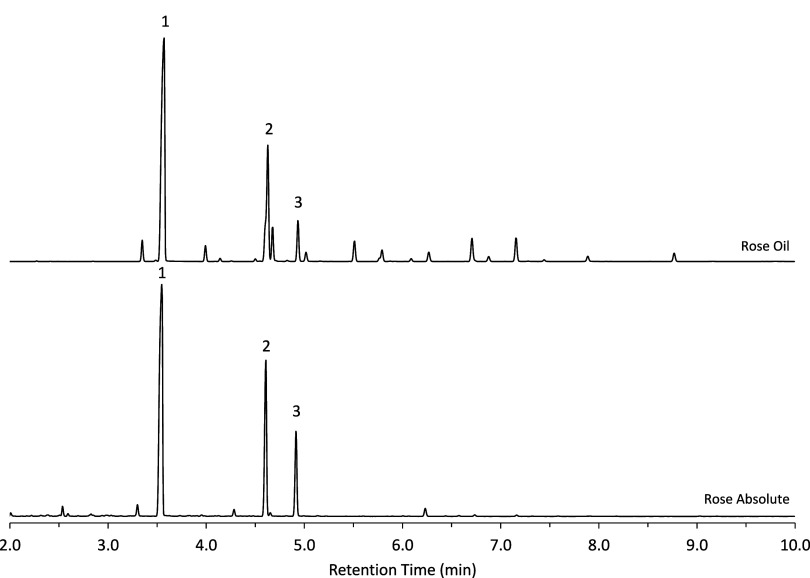
Chromatograms of liquid injection of rose absolute and rose oil. The rose alcohols are identified as 1-PEA, 2-citronellol, 3-geraniol.

The rose perfume sample contained numerous components; however, the three rose alcohols were present at low concentrations and fell outside the calibration range established for each compound (chromatogram not shown, percent areas in Table 3). Any calculated percentages beyond the validated linear range are subject to increased error and should be interpreted accordingly. Despite the variability among commercial samples, GC-MS proved to be effective in quantifying the three rose alcohols, underscoring its value in liquid fragrance analysis.

## Part 2. SPME Headspace Analysis

Sampling the headspace above a natural extract or perfume is advantageous, as only the volatile components in the sample are analyzed and materials can be sampled in situ. Using SPME, the headspace above each standard and commercial sample was analyzed for percent composition and compared to direct liquid injection results. A time-based extraction study was conducted, with two time points illustrated in Figure 5 and Table 4. Within 20 min, the more volatile PEA and citronellol were readily extracted by the SPME fiber, while the least volatile components produced only small peaks. Over time, the remaining six components become more prominent. By the 2 h mark, the peak ratios more closely resembled those observed in the liquid samples (see Figure 1). This shift indicates that the ratio of PEA to the other components changes over time, as equilibrium is not yet established at the 20 min time point.

**Figure 5:**
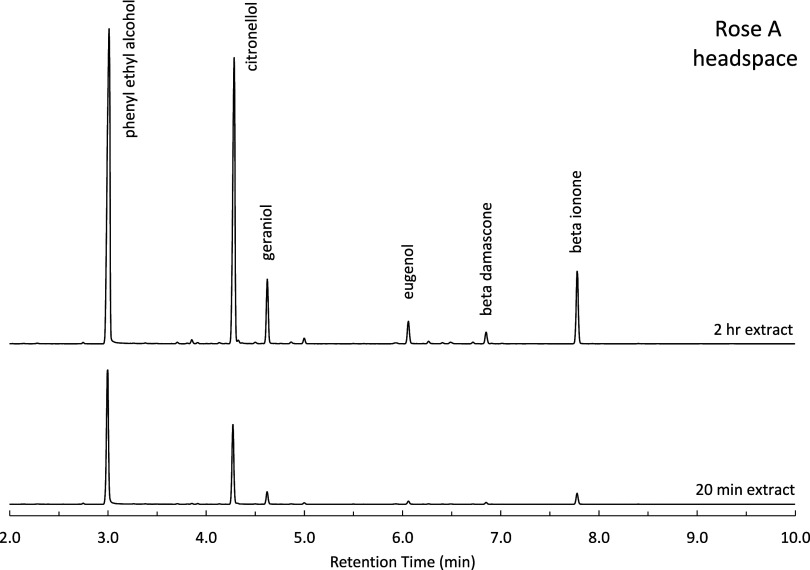
Chromatography of standard rose A using SPME for 20 min and 2 h.

**Table 4:** Peak Areas for Two Headspace Extraction Times Shown in Figure 5

	PEA	citronellol	geraniol	eugenol	β damascone	β ionone
2 h	5.4 × 10^8^	4.1 × 10^8^	7.8 × 10^7^	3.0 × 10^7^	1.5 × 10^7^	9.6 × 10^7^
20 min	1.8 × 10^8^	9.7 × 10^7^	1.5 × 10^7^	4.4 × 10^6^	2.6 × 10^6^	1.5 × 10^7^

Several factors contribute to the low extraction efficiency of the least volatile components. First, differences in volatility play a key role. Larger molecules with lower vapor pressures require more time to enter the vapor phase. Second, in this study, the least volatile components were also present at low concentrations. According to Raoult’s law, components with both low concentration and low vapor pressure will be underrepresented in the headspace relative to the liquid. Third, only one type of SPME fiber was used; alternative fiber chemistries may be better suited for capturing the lower volatility compounds. Lastly, the samples were neither agitated nor heated during extraction, both known factors to accelerate equilibrium. To improve extraction of less volatile components, it is hypothesized that extended extraction time, agitation, or heating would be beneficial, assuming concentration and fiber chemistry remain constant. However, the objective of this study was not to reach equilibrium, but rather to assess whether the percent composition of rose alcohols could be determined using a rapid extraction method comparable in duration to direct liquid injection. Since rose alcohols are the most volatile of the six components in the standard, a shorter extraction time point was appropriate for this analysis.

Peak areas for each standard analyzed by headspace SPME with a 20 min extraction period were determined and are presented in Figure 6. As anticipated given the short extraction duration at ambient temperature, the most volatile compounds, those present at a higher concentration in this study, were rapidly extracted, and represent a greater proportion of the total chromatographic area relative to their proportion in the liquid phase. Conversely, the less volatile compounds contribute a smaller proportion of the total chromatographic area compared to their representation in the liquid phase (see Figure 3 for comparison). Although the absolute peak ratios differ from those obtained via liquid injection, the relative ratios between compounds (e.g., PEA to citronellol) are consistent with the formulation composition. For example, rose standard C contains the highest concentration of PEA, whereas rose standard E exhibits the highest concentration of citronellol (refer to Table 2). Based on these observations, it was hypothesized that a calibration curve based on percent composition could be applied, despite the extraction not reaching equilibrium for PEA, citronellol, and geraniol. To assess method reproducibility, standard B was analyzed in triplicate. The standard deviations calculated for the SPME method ranged from 0.05 to 1.2%, comparable to those obtained via liquid injection.

**Figure 6:**
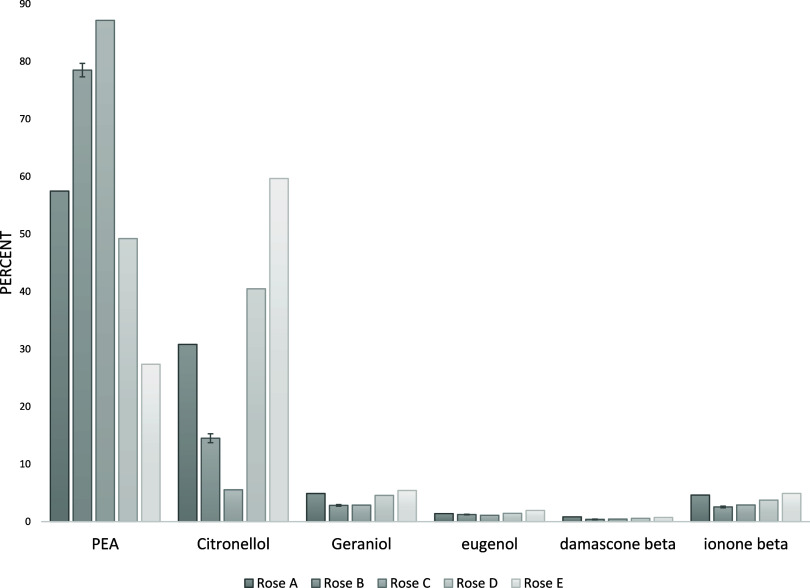
Percent area of each component in the rose standards headspace using SPME with a twenty-minute extraction time.

The percent composition of each rose alcohol in the headspace, as determined via the SPME method, was utilized to construct calibration curves based on percentage values. Standards A through E were employed for PEA and citronellol (12–60%), while standards A, F, and I were used for geraniol (4–15%). The resulting linear regressions are presented in Table 5. The correlation coefficients indicate satisfactory linearity, although slightly lower values were observed for citronellol and geraniol compared to those obtained from the liquid-phase calibration (see Table 2). This deviation may be attributed to incomplete equilibration of analytes during the headspace extraction process.

**Table 5:** Table with Calibration Results for SPME Percent Composition for Each “Unknown” Sample are Provided^a^

	regression	*R*_2_	lab rose	rose absolute	rose oil	peony flower
PEA	*y* = 4.056 × 10^6^ × + 4.760 × 10^7^	0.993	39 ± 1	48	22	--*
citronellol	*y* = 3.587 × 10^6^ × – 3.224 × 10^7^	0.963	33 ± 1	22	15	78*
geraniol	*y* = 1.415 × 10^6^ × + 1.385 × 10^6^	0.962	8.9 ± 0.7	11	3*	10

a Those percent compositions labeled with * fall outside the calibration range of the regression.

Headspace analysis of the commercial samples was conducted using the same 20 min SPME extraction method. Chromatograms for the rose absolute and rose oil are presented in Figure 7, while for the chromatogram for the rose perfume is shown in Figure 8. As observed previously, the rose absolute consists predominately of the three rose alcohols, whereas the rose oil contains a broader array of volatile constituents. Peak intensities and areas obtained via headspace SPME differ from those observed in liquid injection, with early eluting components exhibiting enhanced signals and later eluting components being underrepresented. Nonetheless, the detection of later eluting peaks demonstrates potential for qualitative analysis of less volatile analytes, even under short nonequilibrium extraction conditions. The rose perfume sample yielded a complex chromatographic profile with numerous peaks and relatively low concentrations of the three rose alcohols, consistent with results from liquid-phase analysis. Notably, the application of HS-SPME to a fragrance strip sprayed with perfume represents a novel analytical approach, as it has not been documented in existing literature.

**Figure 7:**
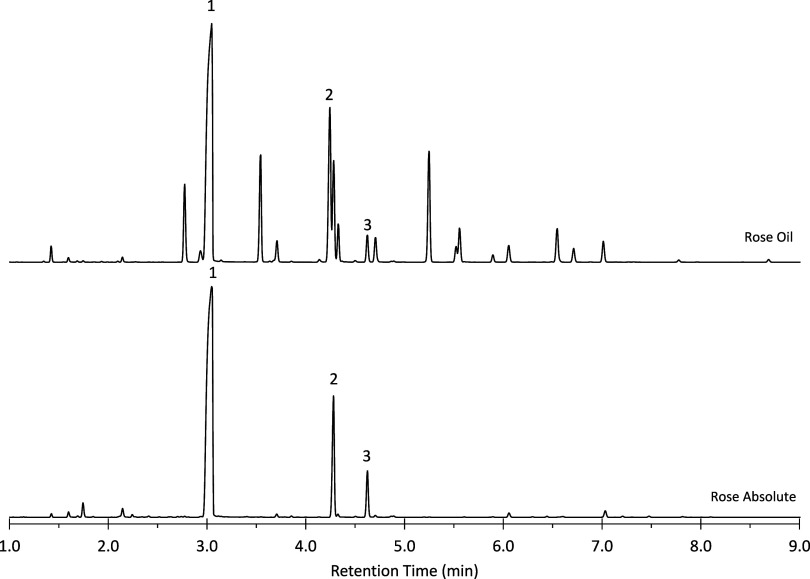
Chromatograms of headspace analysis by SPME of rose absolute and rose oil.

**Figure 8:**
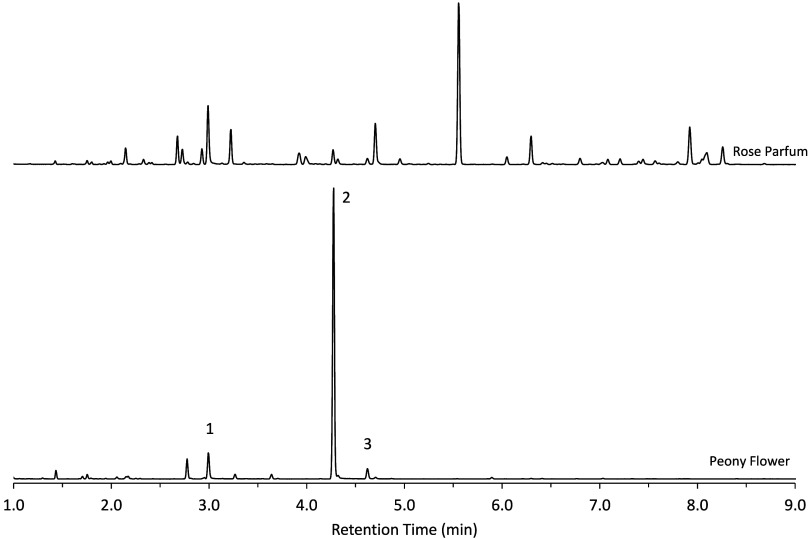
Chromatogram of headspace analysis by SPME of peony flower and rose parfum. The rose alcohols are identified as 1-PEA, 2-citronellol, and 3-geraniol.

The percent composition of each rose alcohol in the commercial samples, calculated using the established calibration data, is shown in Table 5 and Figures 7 and 8. The commercial rose perfume was excluded from the quantitative headspace calibration due to its peak areas falling outside the calibration range for both liquid and headspace analyses. Among the samples, the rose absolute exhibited the closest agreement in calculated component percentages with the liquid injection data, likely due to its composition being dominated by the three rose alcohols and thus minimally affected by the abbreviated extraction time. The laboratory prepared rose sample showed the next highest similarity, with deviations attributed to the presence of three additional volatile compounds that altered the relative peak ratios. In contrast, the rose oil displayed greatest discrepancy in calculated component percentages compared to the liquid-phase data. This error is attributed to the complexity of the sample matrix and the differential volatility of its constituents, which results in disproportionate peak intensities in the headspace analysis. Highly volatile compounds were overrepresented as percentages in the vapor phase, while less volatile compounds were suppressed, leading to skewed total area contributions. These findings underscore the importance of considering extraction time as a critical variable when performing quantitative analysis via HS-SPME.

Standard deviations (*n* = 3) for the lab rose sample analyzed via HS-SPME analysis are reported in Table 5. The absence of variation in standard deviation between the two methods underscores the precision of HS-SPME, affirming its suitability as a robust analytical tool for fragrant compound profiling.

To evaluate the applicability of the headspace SPME method to nonliquid botanical samples, a fragrant peony flower was harvested for analysis. The peony was selected based on its known content of rose alcohols, as well as its seasonal availability. Both the container volume and extraction duration were adjusted to accommodate the physical dimensions of the flower. Headspace analysis of the peony yielded a chromatographic profile dominated by citronellol, with lower concentrations of PEA and geraniol (Figure 8). Although the concentrations of the citronellol and PEA exceeded the calibration limits, geraniol fell within the linear range of its calibration curve (Table 4). These findings demonstrate the potential of headspace SPME, coupled with percent-based calibration, for the analysis of solid botanical samples and intact flowers.

Previous studies have quantified rose alcohols in various rose flower species; however, reported values exhibit substantial variation across samples. Moreover, these studies did not include the production of rose oils or absolutes from the analyzed flowers. Consequently, a direct correlation between the rose alcohol content of the fresh flower and that of the derived essential oil or absolute remains unestablished. Further investigation is warranted to elucidate the impact of extraction and distillation processes on the final composition of rose alcohols. Such research would be instrumental in understanding production-related variability and could aid in the detection of adulteration in commercial essential oils and absolutes.

## Conclusions

This study presents a robust analytical method for the quantitation of three rose alcohols, phenyl ethyl alcohol, citronellol, and geraniol, in natural rose extracts, commercial perfumes, and botanical samples. Liquid injection provided the most accurate representation of the original liquid formulations, serving as a benchmark for comparison. PCA effectively clustered rose accord standards based on the percent composition of rose alcohols and minor chemical constituents, demonstrating its utility in distinguishing sample categories and chemical profiles.

Calibration of the percent area for each rose alcohol was successfully achieved across both natural and synthetic materials without the use of an internal standard. In cases where direct liquid sampling is impractical or the sample is nonliquid, headspace analysis via HS-SPME offers a viable alternative. HS-SPME enabled rapid and effective detection of the target rose alcohols in various commercial products along with additional volatile components. While calibration accuracy decreased with increasing sample complexity, particularly in matrices containing numerous volatile compounds, the method remained capable of estimating rose alcohol percentages and identifying supplementary peaks. Notably, headspace analysis of a fresh peony flower confirmed the presence of all three rose alcohols, underscoring the potential of HS-SPME for analyzing solid botanical samples. Overall, this approach enables the determination of rose alcohol concentrations in both liquid and headspace of diverse sample types. The method offers a promising tool for monitoring compositional changes throughout the production process of rose-derived materials and may aid in the detection of adulteration in essential oils and absolutes.
